# Tetracycline as a Peroxidase Inhibitor: Possible Implications for Thyroid Hormone Biosynthesis

**DOI:** 10.3390/antibiotics15090831

**Published:** 2026-08-27

**Authors:** Sotiris K. Hadjikakou, Christina N. Banti, Kristi Gjerazi

**Affiliations:** Biological Inorganic Chemistry Laboratory, Department of Chemistry, University of Ioannina, 45500 Ioannina, Greece

**Keywords:** biological inorganic chemistry, tetracycline, thyroid dysfunction, thyroid peroxidase inhibition, enzyme kinetics, experimental and computational studies

## Abstract

Tetracycline (TC), a widely used broad-spectrum antibiotic, was experimentally and theoretically investigated for its potential effect on thyroid hormone biosynthesis through interaction with thyroid peroxidase (TPO). Horseradish peroxidase (HRP) was used as a model of TPO, while FeTPPCl was employed as a model of the active site of the enzyme. The inhibitory effect of TC on the catalytic oxidation of iodide to active iodine was studied spectrophotometrically through the formation of triiodide (I_3_^−^), and the IC_50_ value was determined. Kinetic studies using Michaelis–Menten and Lineweaver–Burk analysis demonstrated that TC acts as a reversible non-competitive inhibitor of HRP, leading to decreased Km and Vmax values and the formation of the ESI complex. Molecular docking calculations further supported the experimental findings, indicating that TC binds at a site different from the active center of the enzyme. The results suggest that tetracycline may interfere with thyroid hormone biosynthesis by inhibiting TPO-mediated iodide oxidation, revealing a possible mechanism associated with thyroid-related side effects during prolonged antibiotic administration.

## 1. Introduction

The thyroid gland plays a fundamental role in the regulation of metabolism, growth, and cellular homeostasis through the biosynthesis of the thyroid hormones thyroxine (T4) and triiodothyronine (T3) [1,2,3,4,5,6,7,8,9,10,11]. The biosynthesis of these hormones is initiated by thyroid peroxidase (TPO), a heme-containing enzyme that catalyzes the oxidation of iodide to active iodine in the presence of hydrogen peroxide, followed by iodination of tyrosyl residues of thyroglobulin [12,13,14]. TPO-mediated reactions are therefore essential for the formation of mono- and diiodotyrosine intermediates and ultimately for the production of T3 and T4 [14,15]. Several compounds are known to interfere with this pathway, including clinically used antithyroid drugs such as methimazole (MMI) and propylthiouracil (PTU), which inhibit thyroid hormone biosynthesis through interaction with TPO catalytic pathways and active iodine intermediates [8,16,17].

Tetracycline (TC) is a broad-spectrum antibiotic extensively used for the treatment of Gram-positive and Gram-negative bacterial infections through inhibition of bacterial protein synthesis [18,19]. Besides its antimicrobial properties, tetracycline exhibits strong metal-binding ability and can interact with metalloproteins and redox-active biological systems because of its polydentate coordination environment [20]. Such interactions raise the possibility that tetracycline may also affect heme-containing enzymes involved in endocrine processes, including thyroid peroxidase. Despite the extensive clinical use of tetracycline antibiotics, limited information is available regarding their possible involvement in thyroid hormone biosynthesis and thyroid-related side effects.

Horseradish peroxidase (HRP) is frequently used as a biomimetic model of TPO [11,21]. Both HRP and TPO are heme-containing peroxidases that use H_2_O_2_ as an oxidizing agent and are able to catalyze the oxidation of iodide. This common catalytic activity makes HRP a useful and well-characterized model for studying reactions related to TPO-mediated iodide oxidation. However, important structural differences exist between the two enzymes. HRP is a plant peroxidase, whereas TPO is a mammalian membrane-associated peroxidase with a more complex domain organization. In addition, differences exist in the environment of the heme group and in the substrate-binding regions of the two enzymes. Therefore, HRP does not represent a complete structural model of TPO but provides a useful functional model for investigating peroxidase-mediated iodide oxidation and its possible inhibition [11,21]. In addition, iron tetraphenylporphyrin chloride (FeTPPCl) has been employed as a model of the active site of TPO for mechanistic studies associated with iodide oxidation and thyroid hormone formation [10,22].

**Scheme 1:**
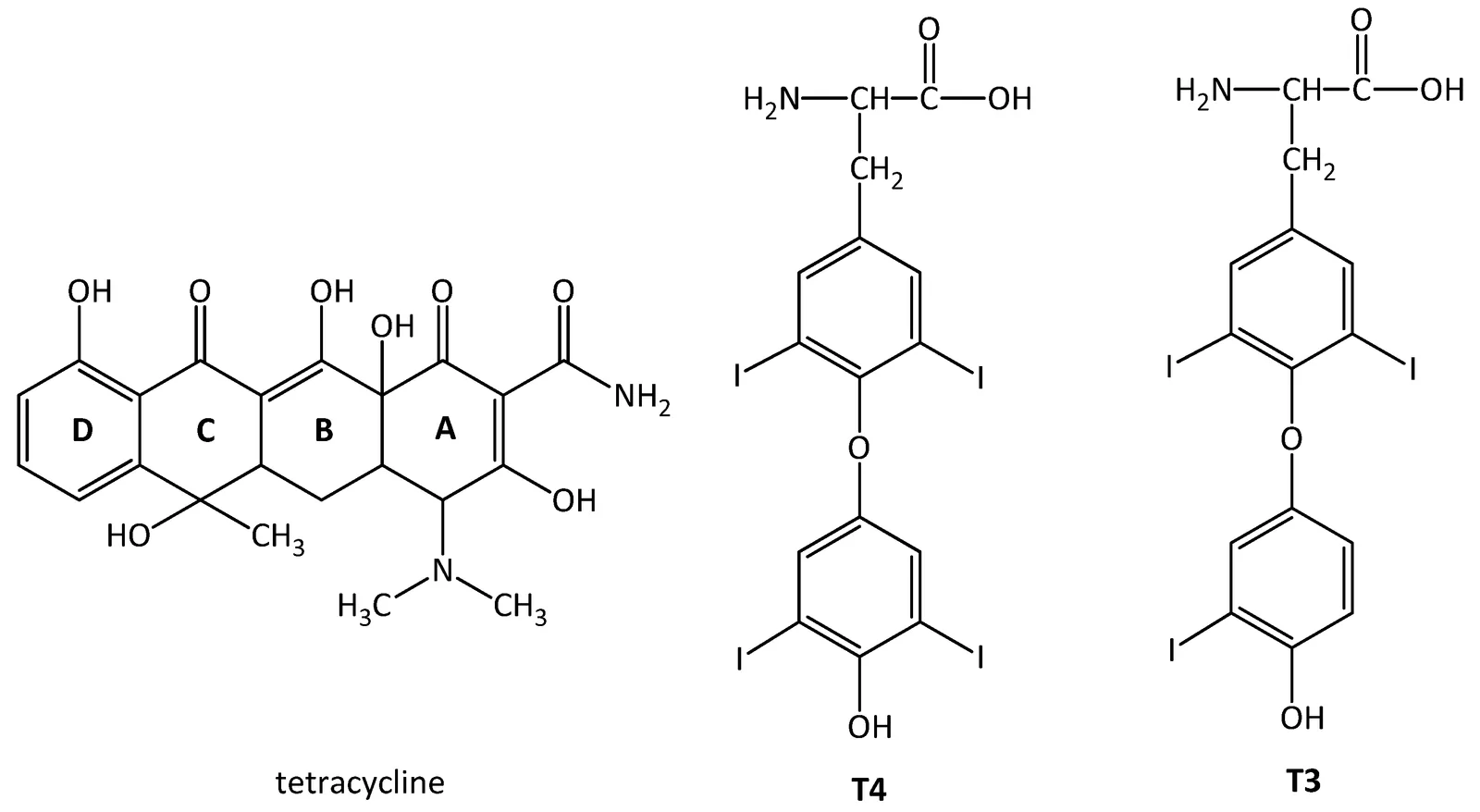
The structure of tetracycline (TC) and thyroid hormones Thyroxine (T4) and Thyronine (T3).

In the course of our studies on the mechanism of action of antithyroid drugs and the side effects that other pharmaceuticals may exert on thyroid hormone biosynthesis [1,2,3,4,5,6,7,8,9,10,11], the interaction of tetracycline (Scheme 1) with HRP and FeTPPCl was investigated both experimentally and theoretically in the present work, in order to elucidate its possible role in the biosynthesis of the thyroid hormones T4 and T3 (Scheme 1). Spectrophotometric kinetic studies, inhibition assays, and molecular docking calculations were performed and compared with the corresponding behavior of methimazole, a clinically established antithyroid drug.

## 2. Results and Discussion

### 2.1. Effect of Tetracycline on Horseradish Peroxidase (HRP) Activity

**Figure 1:**
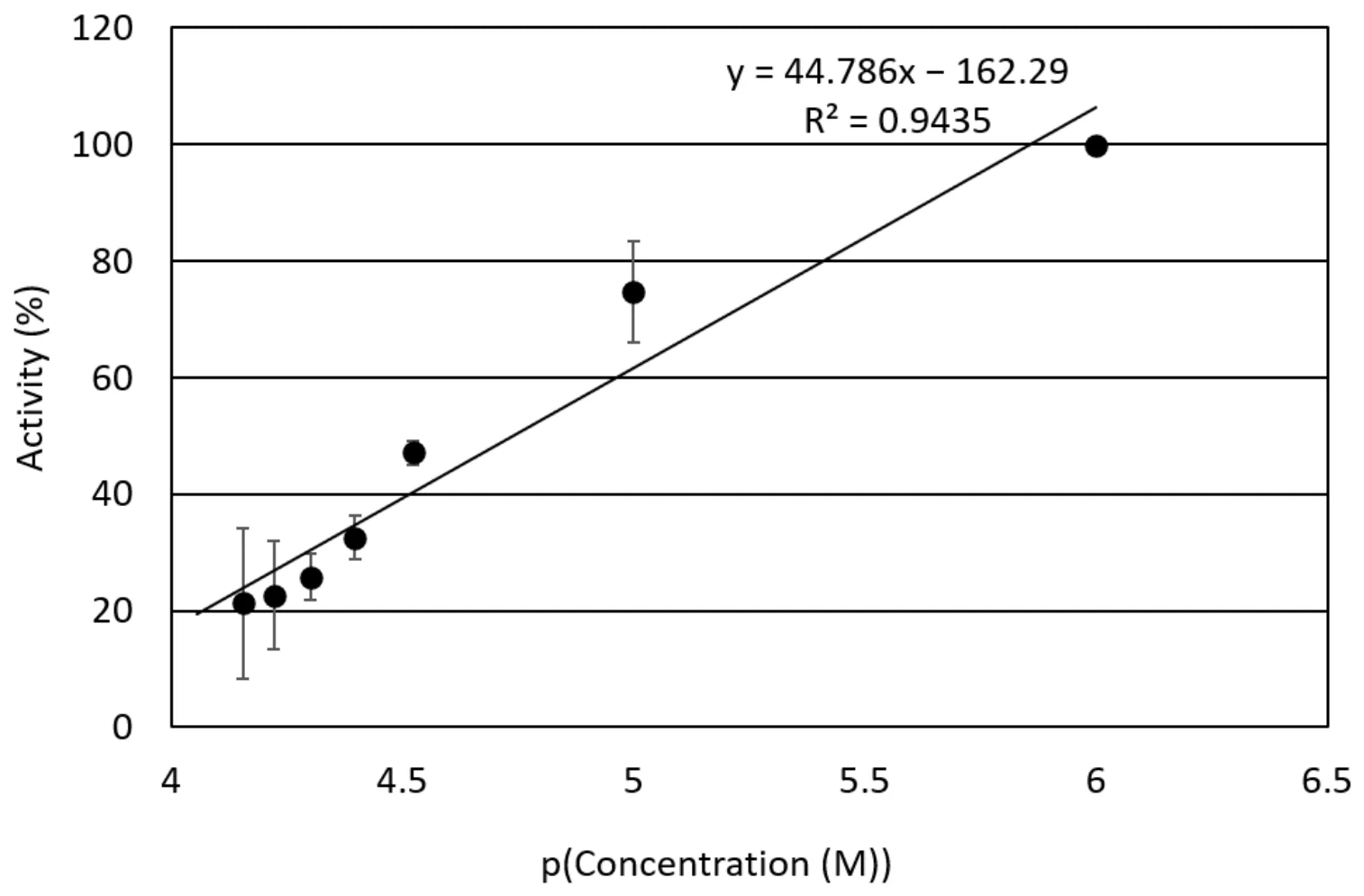
HRP activity (A%) as a function of tetracycline concentration, expressed as pC, where pC = −log C and C is the molar concentration of TC. Data are expressed as mean ± SD from three independent experiments (*n* = 3), and the error bars represent the standard deviation (SD). The pC representation was used for the determination of the IC_50_ value.

*UV–Vis Spectroscopic Study*: The effect of TC on the catalytic activity of HRP, used as a model of TPO, was investigated spectrophotometrically by monitoring the oxidation of iodide to triiodide (I_3_^−^). The enzymatic activity (A%) was determined from the initial reaction rates measured in the absence and presence of increasing concentrations of TC. Figure 1 presents the percent inhibition curve of HRP enzymatic activity vs. −Log(concentration) of TC.

The IC_50_ is defined as the concentration of tetracycline that inhibits 50% of the enzymatic activity. Therefore, from the plot of enzymatic activity (A%) versus pC, the pIC_50_ corresponds to the pC value at which the residual enzymatic activity is 50% of the initial activity. The corresponding IC_50_ value is then calculated according to: IC_50_ = 10^−pIC50^ (M). The IC_50_ value of TC toward HRP is determined to be 36.3 ± 3.2 μM. The reference antithyroid drugs methimazole (MMI) and propylthiouracil (PTU) exhibited greater inhibitory potency, with IC_50_ values of 18.9 μM and 27.8 μM, respectively [11]. Thus, TC interacts with HRP, reducing its catalytic activity and suggesting a potential interference with thyroid peroxidase (TPO)-like enzymatic function. To further elucidate the inhibitory mechanism of TC, enzyme kinetic studies, molecular docking analyses, and catalytic activity assays using the iron–porphyrin model system FeTPPCl, which mimics the heme-containing active site of HRP, were performed.

**Figure 2:**
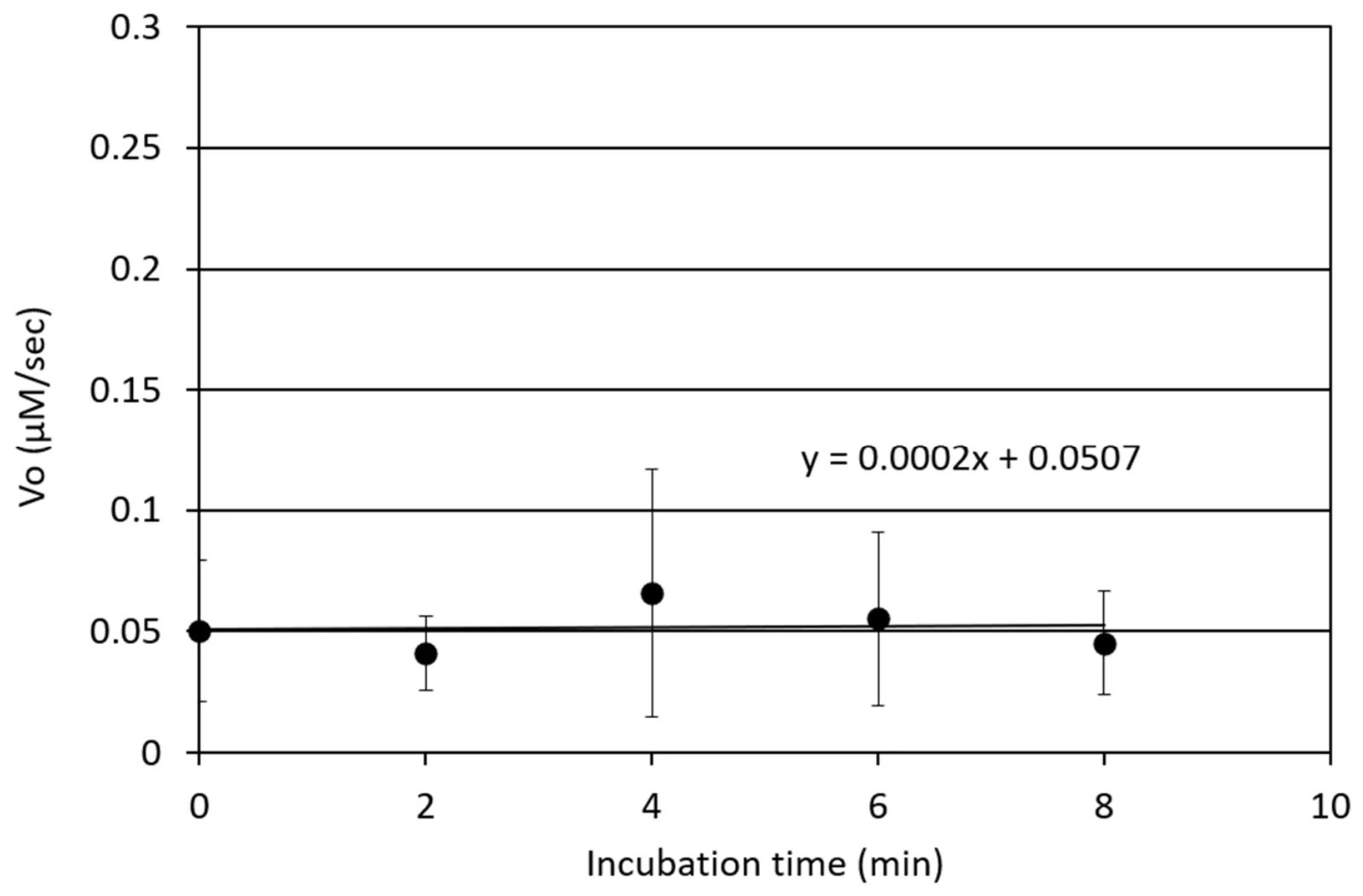
V_o_ (μΜ/s) vs. difference in incubation interval time (min) upon tetracycline.

To further investigate the mechanism of inhibition, the enzyme HRP was pre-incubated with TC for different time intervals (Figure 2). The V_o_ values at all incubation time intervals remained unchanged, suggesting that TC is a reversible inhibitor, similar to MMI and PTU [11].

### 2.2. Type of Inhibition Mechanism

**Figure 3:**
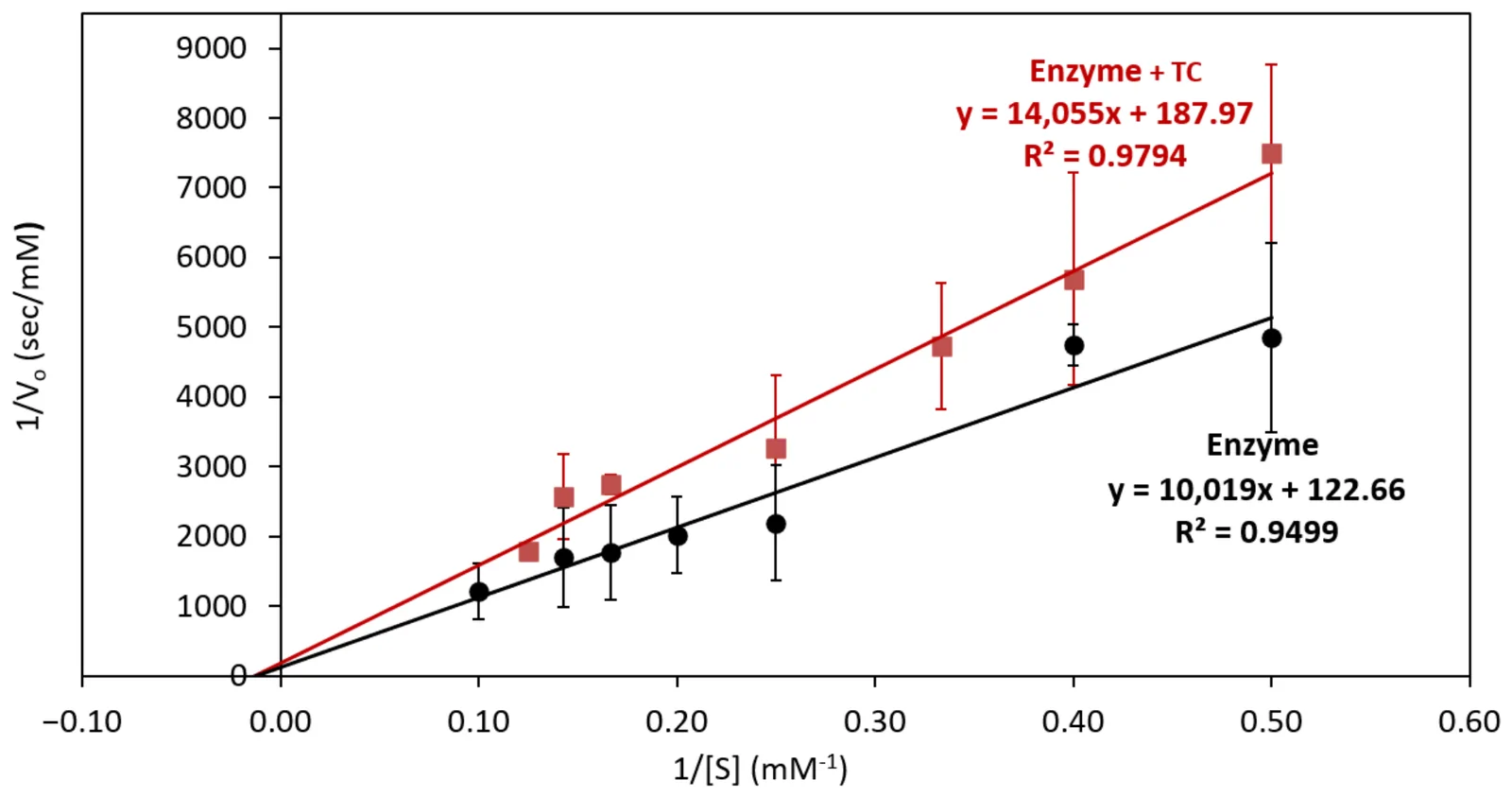
Lineweaver–Burk plots for the determination of K_M_ and V_max_ of HRP in the absence and presence of TC. Data are expressed as mean ± SD from three independent experiments. Error bars represent the standard deviation (SD). The corresponding linear regression equations and R^2^ values are shown in the graph.

The inhibitory mechanism of TC was further investigated by steady-state kinetic analysis over a range of substrate concentrations (0.5–5 mM for the control and 0.5–6 mM in the presence of TC). Enzyme kinetics were evaluated in both the absence and presence of the inhibitor, and the experimental data were analyzed using Lineweaver–Burk double-reciprocal plots (Figure 3). The kinetic parameters, K_M_ and V_max_, were determined from the slope and intercept of the corresponding linear regression plots from the Lineweaver–Burk Equation (1):

(1) $$\frac{1}{V_{0}}=\frac{K_{M}}{V_{max}}\frac{1}{\left[S\right]}+\frac{1}{V_{max}}$$

The Lineweaver–Burk double-reciprocal plots (Equation (1) and Figure 3) yielded the following linear regression equations: y = 10,019x + 122.66 (R^2^ = 0.95) for the control experiment and y = 14,055x + 187.97 (R^2^ = 0.98) in the presence of TC. The kinetic parameters obtained from three independent experiments were K_M_ = 81.7 mM and V_max_ = 8.2 × 10^−3^ mMs^−1^ for HRP in the absence of TC, and K_M_ = 74.8 mM and V_max_ = 5.3 × 10^−3^ mMs^−1^ in the presence of TC. Thus, in the presence of TC, the V_max_ value decreased by 35.4%**,** whereas the K_M_ value decreased by only 8.4% compared with the control experiment. The much higher reduction in V_max_ value than K_M_ indicates that TC primarily affects the catalytic activity of HRP, while having only a limited effect on substrate binding. These results are consistent with a predominantly non-competitive inhibition mechanism. In a non-competitive inhibition mechanism, TC binds to both the free enzyme (E) and the enzyme–substrate complex (ES), leading to the formation of EI and ESI complexes. The larger reduction in V_max_ than in K_M_ indicates that TC mainly affects the catalytic activity of HRP rather than substrate binding.

### 2.3. In Silico Studies

**Figure 4:**
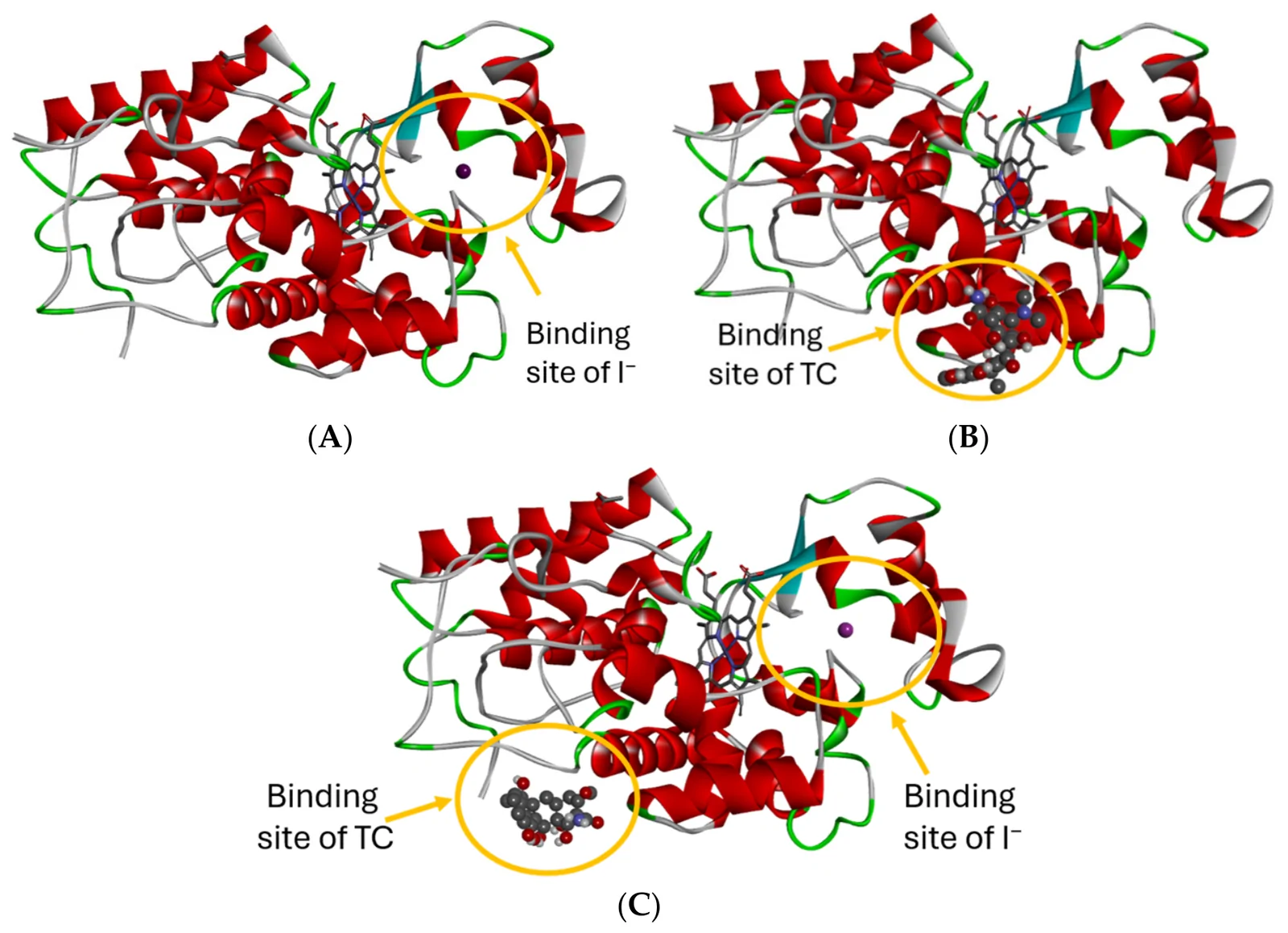
Binding sites of ES (**A**), EI (**B**), and ESI (**C**).

In order to investigate the binding mode of TC to HRP, molecular docking studies were performed (Figure 4). The crystal structure of the unbound enzyme (PDB ID: 1H5G) was used as the receptor. The crystal structure of the unbound enzyme (PDB ID: 1H5G) was used as the receptor. This structure was selected because the unbound form of HRP allows the investigation of ligand binding without assuming a specific binding site. The docking search space included the entire enzyme, allowing TC to explore different possible binding sites. This approach was considered appropriate because the kinetic studies suggested a predominantly non-competitive inhibition mechanism, in which TC may bind at a site different from the substrate-binding site. Therefore, the docking studies were performed to investigate the possible binding site of TC and to support the experimental kinetic results. In addition, the use of PDB ID 1H5G is consistent with our previous computational studies using HRP [11].

Table 1 summarizes the ES, EI, and ESI binding energies, as well as the amino acid (AA) residues that form the docking cavity of the substrate and/or the inhibitor and the distance (d) of the substrate and/or the inhibitor from the close amino acid residues of the cavity (AA). The binding energy of HRP with triiodide (I_3_^−^) was calculated to be −1.04 kcal mol^−1^, whereas the binding energy of the HRP-TC (EI) complex was −5.96 kcal mol^−1^. The binding mode of TC was further investigated using the enzyme–substrate–inhibitor (ESI) complex. The calculated binding energy of the HRP-I_3_^−^-TC (ESI) complex was −6.50 kcal mol^−1^, which was lower than that of the HRP-TC (EI) complex (−5.96 kcal mol^−1^), indicating a more stable interaction. In both the EI and ESI complexes, TC binds at a site distinct from the substrate-binding site, indicating that substrate binding is not prevented (Table 1).

These results are consistent with a non-competitive inhibition mechanism and are in good agreement with the steady-state kinetic analysis. In a non-competitive inhibition mechanism, the inhibitor binds to both the free enzyme (E) and the enzyme-substrate complex (ES), leading to the formation of the EI and ESI complexes. A non-competitive inhibitor binds to a site different from the catalytic site of the enzyme. Therefore, it does not compete with the substrate for binding. Instead, it changes the conformation of the enzyme and reduces its catalytic activity, while substrate binding remains largely unaffected [10,11,23,24,25].

### 2.4. Inhibition of the Catalytic Activity of FeTPPCl

Since TPO contains an iron–porphyrin active site that catalyzes the oxidation of iodide to active iodine in the presence of hydrogen peroxide, followed by the iodination of tyrosyl residues in thyroglobulin, we extended our studies to investigate the reactivity of tetracycline toward iodide oxidation in the presence of FeTPPCl, which serves as a model of the TPO active site. The iodide anion (I^−^) is readily oxidized by H_2_O_2_ through a two-electron transfer process, either non-enzymatically or enzymatically in the presence of peroxidases, according to the following equation [10]:

(2) $$2I^{-}+H_{2}O_{2}\to I_{2}+H_{2}O$$

(3) $${I^{-}+I}_{2}⇌I_{3}^{-}$$

Oxidation reactions of iodide anion by hydrogen peroxide.

The inhibition activity of TC was measured in the presence of FeTPPCl (a model of the active site of TPO) and H_2_O_2,_ yielding I_3_^−^ resulting from the oxidation of I^−^ according to reaction (2).

**Figure 5:**
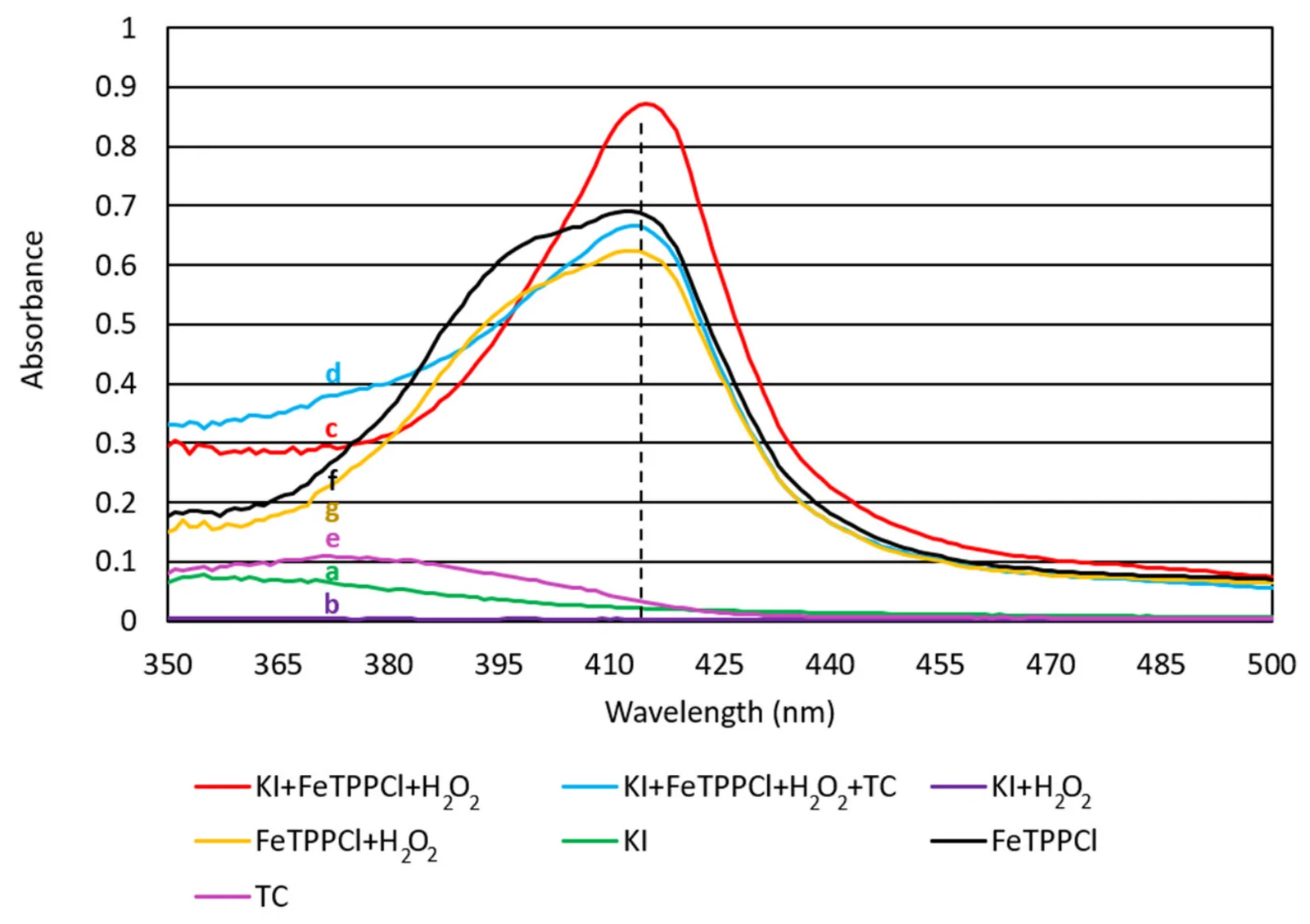
UV spectra of the effect caused by TC on the catalytic activity of FeTPPCl.

The final rSeaction product monitored was triiodide (I_3_^−^), which exhibits a characteristic UV-Vis absorption band at 415 nm. Figure 5 presents the UV-Vis spectra of DMSO solutions of: (a) KI (10 mM); (b) KI (10 mM) and H_2_O_2_ (50 μM); (c) KI (10 mM), H_2_O_2_ (50 μM), and FeTTPCl (5 μM); and (d) KI (10 mM), H_2_O_2_ (50 μM), FeTTPCl (5 μM), and TC (5 μM). For comparison, the spectra of (e) TC (5 μM), (f) FeTTPCl (5 μM), and (g) FeTTPCl (5 μM) in the presence of H_2_O_2_ (50 μM) are also included.

The UV-Vis spectra indicate that the FeTPPCl/H_2_O_2_/KI system catalyzes the oxidation of iodide, leading to the formation of triiodide (I_3_^−^), as evidenced by the presence of an absorption band at 415 nm. Upon addition of TC, the absorbance at 415 nm decreased, indicating a reduced formation of I_3_^−^ and inhibition of the catalytic oxidation of iodide. Thus, TC suppresses the catalytic activity of the FeTPPCl/H_2_O_2_ system.

However, the UV-Vis spectra alone do not provide direct evidence that tetracycline binds to the catalytic heme center (active site) of the enzyme model. The reduced I_3_^−^ formation may arise from several mechanisms, including inhibition of the catalytic cycle, interaction with reactive intermediates (Compound I or Compound II), or scavenging of oxidizing species. Therefore, these spectral data should be regarded as evidence of reduced catalytic activity, rather than definitive proof of active-site binding.

## 3. Conclusions

The present study provides experimental and computational evidence that tetracycline can interfere with peroxidase-mediated iodide oxidation. TC inhibited HRP activity in a concentration-dependent and reversible manner, while the kinetic studies indicated a predominantly non-competitive inhibition mechanism. Molecular docking studies further supported the experimental findings, showing that TC binds to HRP at a site different from the substrate-binding site. In addition, TC reduced the catalytic activity of the FeTPPCl model system; however, these results do not support a direct interaction of TC with the heme iron center.

Therefore, the present findings reveal a previously unexplored interaction of tetracycline with peroxidase systems and suggest a possible mechanism by which this widely used antibiotic may interfere with processes involved in thyroid hormone biosynthesis.

## 4. Experimental Section

*Materials and instruments:* All solvents were of reagent grade and were used as received. Horseradish peroxidase, tetracycline, ferric tetraphenylporphyrin chloride, potassium iodide, and hydrogen peroxide (Sigma-Aldrich, Merck, Burlington, MA, USA) were also used without further purification. Electronic absorption spectra were recorded using a UV-1600 PC series VWR spectrophotometer (Darmstadt, Germany).

### 4.1. Horse Radish Peroxidase (HRP) Inhibition Assay and Molecular Docking Studies

HRP inhibition was evaluated spectrophotometrically following a modified literature protocol. All experiments were performed in 0.050 M sodium acetate buffer (pH = 5.5), prepared by dissolving sodium acetate in double-distilled water, adjusting the pH with 50% (*w*/*v*) NaOH and 37% (*v*/*v*) hydrochloric acid, and diluting to the desired final volume. The substrate solution was freshly prepared by dissolving 1.7 mM KI in dd water. Next, 50 nM horseradish peroxidase (HRP) in PBS buffer and 10 mM H_2_O_2_ in 50 mM ice-cold sodium acetate buffer (pH 5.5) were freshly prepared and maintained on ice throughout the experiments to preserve enzymatic activity. The formation of triiodide was monitored spectrophotometrically at 353 nm (ε = 25,500 M^−1^ cm^−1^) using a UV–Vis spectrophotometer thermostated at 25 °C, in the presence or absence of tetracycline (TC), with a final reaction volume of 2 mL. TC was evaluated over a concentration range of 1–70 μM in DMSO solutions.

The inhibitory mechanism of TC was investigated by steady-state kinetic analysis using KI concentrations ranging from 0.5 to 6 mM, while maintaining constant concentrations of HRP (50 nM) and H_2_O_2_ (10 mM), in the absence and presence of TC at its IC_50_ concentration. The kinetic parameters, K_m_ and V_max_, were determined from the slope and intercept of the Lineweaver–Burk double-reciprocal plots.

All experiments concerning HRP inhibition and the investigation of the inhibition mechanism were performed independently in triplicate, and the kinetic parameters are expressed as mean ± SD.

### 4.2. Docking Studies

AutodockVina was used for the molecular docking studies [26]. The graphical interface AutoDockTools (ADT, version 1.5.7) was used for receptor and ligand preparation [27]. The enzyme structure was treated as rigid, while the ligands were allowed full conformational flexibility without the application of torsional restraints. Prior to docking, all water molecules and crystallographic solvent molecules were removed from the protein structure. The docking search space was defined using a grid box large enough to encompass the entire enzyme, allowing unrestricted exploration of potential binding sites by the ligands. All docking calculations were performed using the default software parameters. The crystal structure of the enzyme (PDB ID: 1H5G) was used, following the procedure previously described in Ref [11]. The output graphics, pocket amino acid residues, and the binder-enzyme distances were drawn by Discovery Studio 2001.

### 4.3. Inhibition of the Catalytic Activity of FeTPPCl

The reaction mixture consisted of 1 mM KI, 1 mM H_2_O_2_, 5 μM FeTPPCl, and 5 μM TC in DMSO. The formation of I_3_^−^ was monitored spectrophotometrically by measuring the absorbance at 415 nm.

## Figures and Tables

**Table 1:** Binding energy values when the substrate (I_3_^−^) binds to the enzyme HRP with or without the presence of TC ES, as well as the amino acid (AA) residues forming the docking cavity of the substrate and/or the inhibitor and the distance (d) of the binder (substrate and/or the inhibitor) from the close amino acid residues of the cavity (AA).

	Binding Energy (kcal mol^−1^)	Amino Acid Residues Form the Docking Cavity of the Substrate and/or the Inhibitor	d(Bider-AA) Å Binder = I_3_^−^, TC or I_3_^−^/TC
HRP-I_3_^−^	−1.04	Arg224, Lys241	Arg224 = 3.67, Lys241 = 3.71
HRP-TC	−5.96	Asp150, Asn137, Ser151, Asn154, Ala136,	Asp150 = 1.95 and 3.72, Asn137 = 2.01, Ser151 = 3.67 and 3.65, Asn154 = 2.21 and 3.59, Ala136 = 2.09
HRP-I_3_^−^-TC	−6.50	Arg283, Leu2, Asn286, Gln1	Arg283 = 2.79, Leu2 = 1.93, Asn286 = 2.19 and 3.40, Gln1 = 4.98

## Data Availability

The data supporting the findings of this study are available from the corresponding authors upon request.
